# Tumor burden is a potential marker of PARP inhibitor effects in ovarian cancer: a head-to-head observational series

**DOI:** 10.1186/s13048-020-00629-4

**Published:** 2020-03-17

**Authors:** Jing Ni, Rui Zhou, Xianzhong Cheng, Xia Xu, Wenwen Guo, Xiaoxiang Chen

**Affiliations:** 1 Department of Gynecologic Oncology, The Affiliated Cancer Hospital of Nanjing Medical University, Jiangsu Cancer Hospital, Jiangsu Institute of Cancer Research, 42# Baiziting street, Nanjing, Jiangsu 210009 People’s Republic of China; 2 Department of Chemotherapy, The Affiliated Cancer Hospital of Nanjing Medical University, Jiangsu Cancer Hospital, Jiangsu Institute of Cancer Research, 42# Baiziting street, Nanjing, Jiangsu 210009 People’s Republic of China; 3 Department of Pathology, The Second Affiliated Hospital of Nanjing Medical University, Nanjing, Jiangsu 210011 People’s Republic of China

**Keywords:** Recurrent ovarian cancer, Olaparib, Tumor burden, Potential marker

## Abstract

**Background:**

Olaparib, a poly ADP-ribose polymerase (PARP) inhibitor, has proven to be effective and safe as maintenance therapy and multiline therapy in ovarian cancer, especially in patients with BRCA mutations. This study intended to observe the influence of tumor load on the efficacy and safety of olaparib in recurrent ovarian cancer.

**Cases presentation:**

Three patients harbored gBRCAwt with low tumor load (LTL), while two women harbored BRCAmt with high tumor load (HTL) were recruited. Two of the three LTL patients achieved partial response, and the other showed stable disease. Both HTL patients were assessed to have progressive disease in a short time. Olaparib appears to be effective and safe for LTL recurrent ovarian cancer patients even if it is gBRCAwt, while the response is poor in HTL patients.

**Conclusions:**

Tumor load may be another potential marker to predict the effect of PARP inhibitors. The present head-to-head observational series provides new evidence on this issue for further research from bench to bedside in the future.

## Introduction

Ovarian cancer is the highest mortal gynecological malignant tumor, while the five-year survival rate has long been teetering at 30% [1]. Currently, the standard treatment for ovarian cancer is maximal cytoreductive surgery and platinum-based chemotherapy [2]. Eighty percent of ovarian cancers recur within 2 years of the initial treatment. Patients with platinum-free interval (PFI) over 6 months are thought to have platinum-sensitive relapsed (PSR) ovarian cancer. The primary treatment of PSR ovarian cancer is still secondary cytoreductive surgery and/or platinum-based chemotherapy [3].

PARP is essential for the repair of single-strand DNA breaks (SSDBs) in the base excision process, and PARP inhibitors (PARPi) can induce synthetic lethality in tumors with homologous recombination deficiency due to the transitions from SSDBs to double-strand DNA breaks (DSDBs) [4]. Olaparib (Lynparza®, AstraZeneca) is an oral PARP inhibitor. Nowadays, clinical trials have confirmed that PARPi as first-line or second-line maintenance therapy significantly increase progression-free survival in ovarian cancer patients with a BRCA1/2 mutation [5, 6]. In addition to maintenance therapy, olaparib can also be used for monotherapy of gBRCA-mutated ovarian cancer after third-line chemotherapy [7]. Recent studies showed that women with gBRCAmt platinum-sensitive recurrent ovarian cancer after second-line chemotherapy [8] and even gBRCAmt platinum-resistant patients [9] could benefit from olaparib monotherapy. An overall survival (OS) advantage was observed with olaparib for PSR ovarian cancer patients irrespective of BRCA1/2 mutation status in the updated survival data of Study19, while the median OS in BRCAmt ovarian cancer was longer than that in the BRCAwt subgroups [10]. These results suggest that BRCAmt ovarian cancer is more likely to benefit from olaparib than BRCAwt ovarian cancer. Other than BRCA pathologic mutations, homologous recombination deficiency and platinum sensitivity, are there any other markers associated with PARP inhibitor effects in ovarian cancer? Here, we observed that the short-term efficacy of PARP inhibitors was influenced by tumor burden.

## Cases presentation

**Fig. 1:**
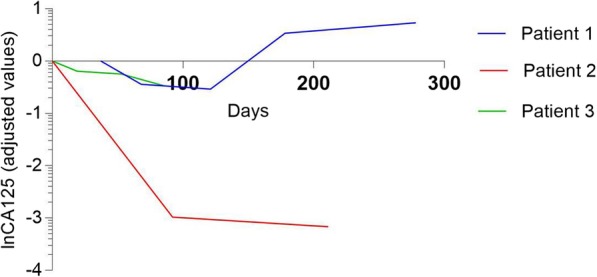
Serum CA125 level in three patients with low tumor load. (The baseline of CA125 was used as the reference value, and all data were converted to natural logarithm.)

**Fig. 2:**
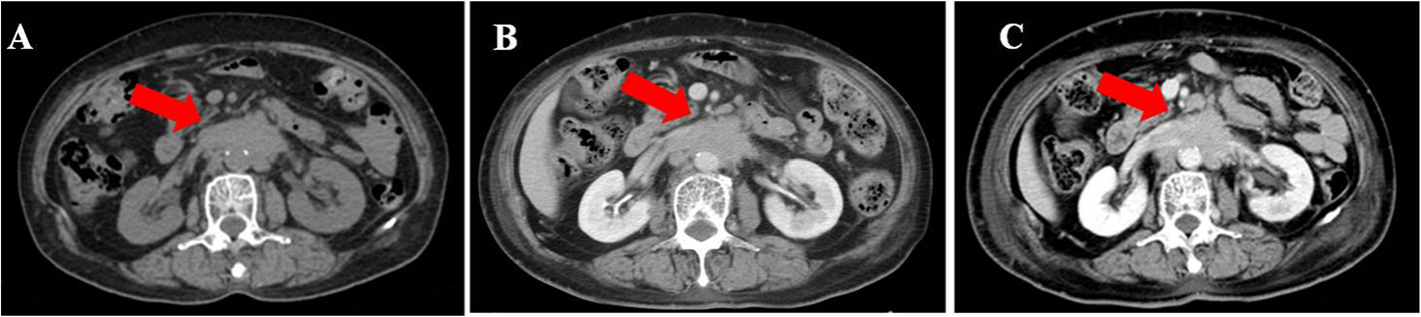
Abdominal CT showing the metastatic para-aortic lymph node. Notes: **a** pretreatment of Olaparib. **b** Six months posttreatment of Olaparib. **c** Nine months posttreatment of Olaparib

Patient 1 was a 73-year-old female noted to have a right-sided ovarian mass by ultrasonography during a routine examination. After the cytoreduction to no macroscopic residual disease (total abdominal hysterectomy, bilateral salpingo-oophorectomy, omentectomy, appendectomy, bilateral pelvic and para-aortic lymph node dissection, metastases excised in the uterus-rectum-fossa), she was diagnosed with stage IIIc high-grade serous papillary adenocarcinoma. Then, she was given 6 cycles of paclitaxel (135 mg/m^2^) and carboplatin (AUC = 5) and achieved complete clinical remission (CR) by computed tomography (CT) and the tumor marker CA125. Approximately 65 months later, her CA125 serum concentration increased to 171.6 U/ml, and a metastatic para-aortic lymph node with a short diameter of 4 cm at the renal hilum level was found by CT (Fig. 2a). The patient was considered to be gBRCAwt PSR ovarian cancer, and secondary cytoreductive surgery and platinum-based chemotherapy were recommended by a multidisciplinary team (MDT). However, she refused our proposal because of her religious belief and took olaparib (150 mg orally twice daily) on her own. Two months later, she came to our centre for routine follow-up, and the CA125 level had decreased to 99.38 U/ml. Routine blood tests found a slight decrease in hemoglobin. In terms of treatment-emergent adverse events (TEAEs), the patient appeared slightly fatigued and had decreased appetite. Unfortunately, the patient developed persistent fever due to erysipelas and stopped taking olaparib for 21 days in the fifth month. Then, she took olaparib at a daily oral dose of 150 mg for 1 month. CT showed that the metastatic para-aortic lymph node shrank to 2.2 cm in short diameter (Fig. 2b), and the other non-target lesions remained similar in size. In the following 3 months, she took olaparib at a daily oral dose of 150 mg twice per day because the serum CA125 was increasing (Fig. 1). The short diameter of metastatic lymph node at the renal hilum was then found by CT to slightly increase to 2.77 cm (Fig. 2c), and the rest remained at a similar size.

**Fig. 3:**
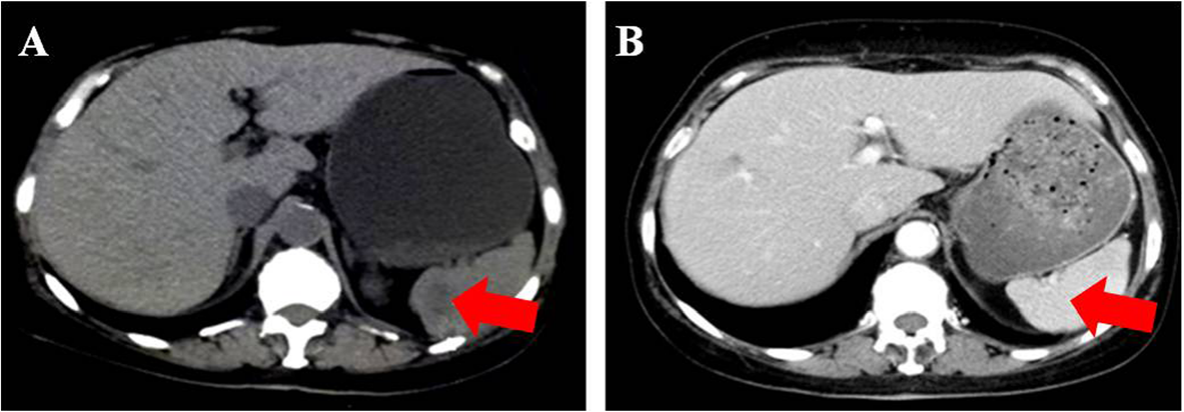
The tumor (arrow) in spleen disappeared. Notes: **a** pretreatment of Olaparib. **b** posttreatment of Olaparib

Patient 2 was a 61-year-old female who presented with intermittent lower abdominal pain with a large cystic and solid mass in the right ovary by ultrasonography. She underwent the cytoreduction to no macroscopic residual disease (total abdominal hysterectomy, bilateral salpingo-oophorectomy, omentectomy, appendectomy, bilateral pelvic and para-aortic lymph node dissection, multi-point biopsy of peritoneal) and was diagnosed with stage IIIa_2_ ovarian carcinosarcoma by pathologic staging. The patient strongly objected to receiving adjuvant chemotherapy due to personal preference. Thirteen months after surgery, she experienced recurrence. Accompanied by an increasing serum CA125 level (94.79 U/ml), there were multiple low-density shadows (the largest shadow was approximately 2.5 cm in size) in the spleen (Fig. 3a), and the liver was thought to have metastatic lesions, with a cystic mass on the right side of the pelvic cavity that was regarded as a lymphatic cyst by CT. Meanwhile, genetic testing showed that she harbored gBRCAwt disease. The patient refused chemotherapy again and took olaparib at a daily oral dose of 300 mg twice per day. She suffered mild arthralgia and anemia in the first month. Seven months after taking the medicine, the serum CA125 level decreased to 4.01 U/ml (Fig. 1), and CT showed that multiple metastases in the spleen disappeared (Fig. 3b). Multiple nodules in the liver were the same as before, and the largest nodule was approximately 1.96 × 1.66 cm in size.

**Fig. 4:**
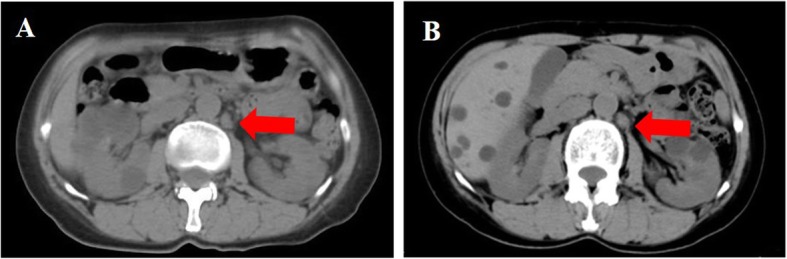
The metastatic para-aortic lymph node pointed with arrow. Notes: **a** pretreatment of Olaparib. **b** posttreatment of Olaparib

Patient 3 was a 51-year-old female with irregular bloody vaginal discharge, and a right-sided pelvic mass was found by ultrasonography. She was confirmed to have gBRCAwt disease. She also received the cytoreduction to no macroscopic residual disease (total abdominal hysterectomy, bilateral salpingo-oophorectomy, omentectomy, appendectomy, bilateral pelvic and para-aortic lymph node dissection, multi-point biopsy of peritoneal) and was diagnosed with stage IIIa_1_ high-grade serous fallopian tube cancer. The patient refused adjuvant chemotherapy after the operation because of leukopenia and was assessed to be in CR by positron emission tomography-computed tomography (PET-CT). After 13 months of follow-up, the serum CA125 concentration increased to 35.6 U/ml, and multiple enlarged lymph nodes with abnormal FDG accumulation, the largest of which was approximately 1.2 × 0.6 cm in size around the abdominal aorta and inferior vena cava, were observed by PET-CT (Fig. 4a). She refused secondary cytoreductive surgery and/or platinum-based chemotherapy and took olaparib (150 mg orally twice daily) on her own. While she was taking olaparib, the CA125 level decreased gradually every month (Fig. 1). Three months later, the largest short diameters of the lymph nodes beside the abdominal aorta were approximately 7 mm by CT (Fig. 4b). The TEAEs included mild fatigue, anemia and abdominal pain from the second month.

**Fig. 5:**
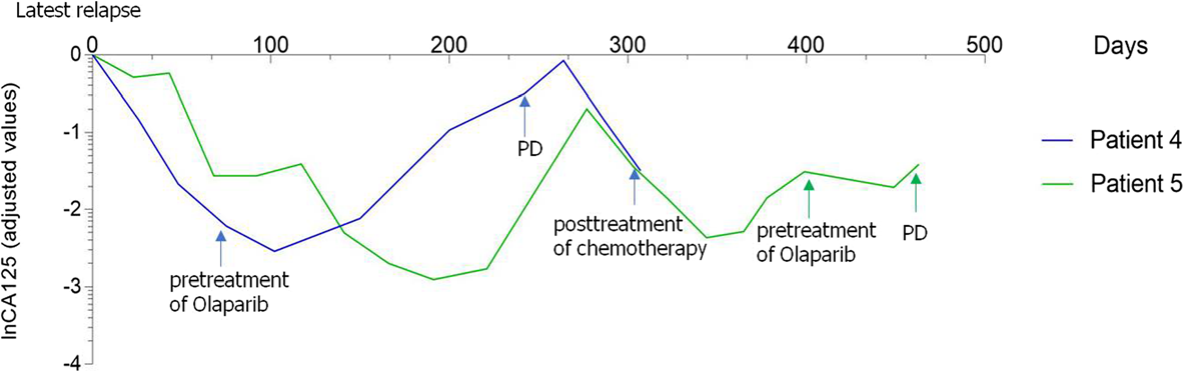
Serum CA125 level in two patients with high tumor load. (The baseline of CA125 was used as the reference value, and all data were converted to natural logarithm)

**Fig. 6:**
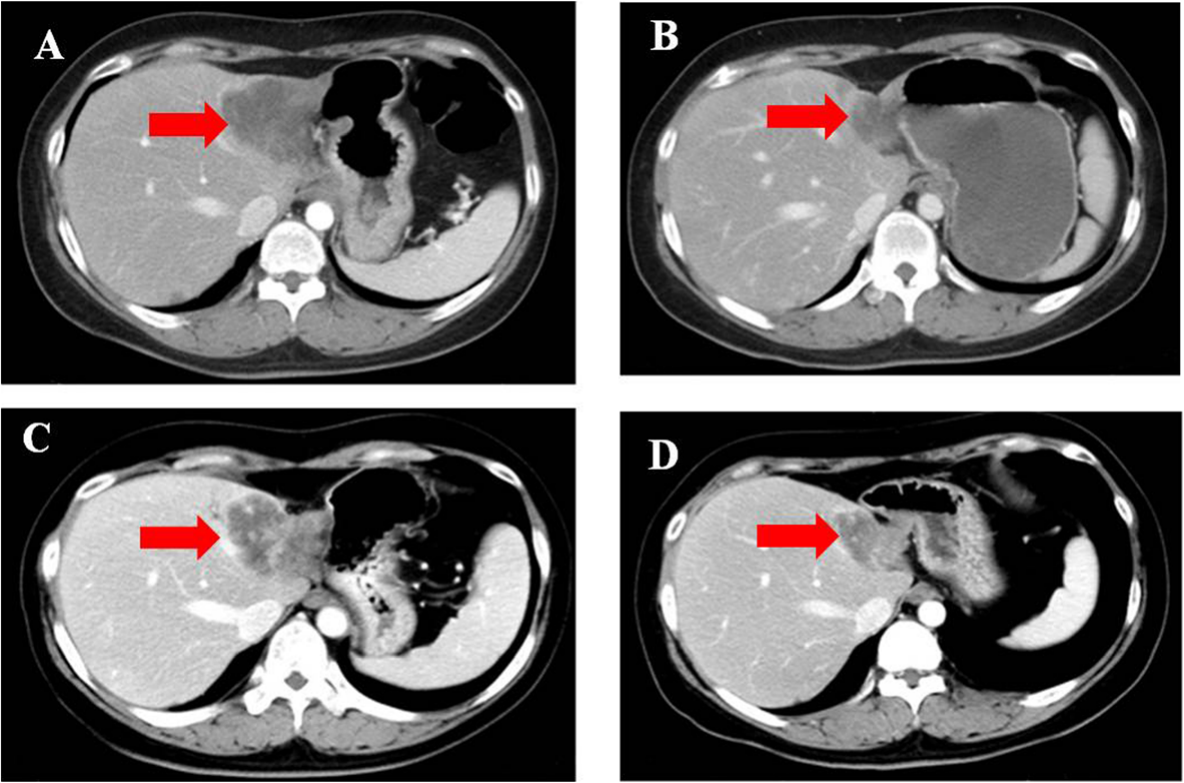
The hepatic metastatic pointed with arrow. Notes: **a** recurrence before chemotherapy. **b** partial response after chemotherapy/pretreatment of Olaparib. **c** progressive disease after using Olaparib. **d** partial response after chemotherapy

**Fig. 7:**
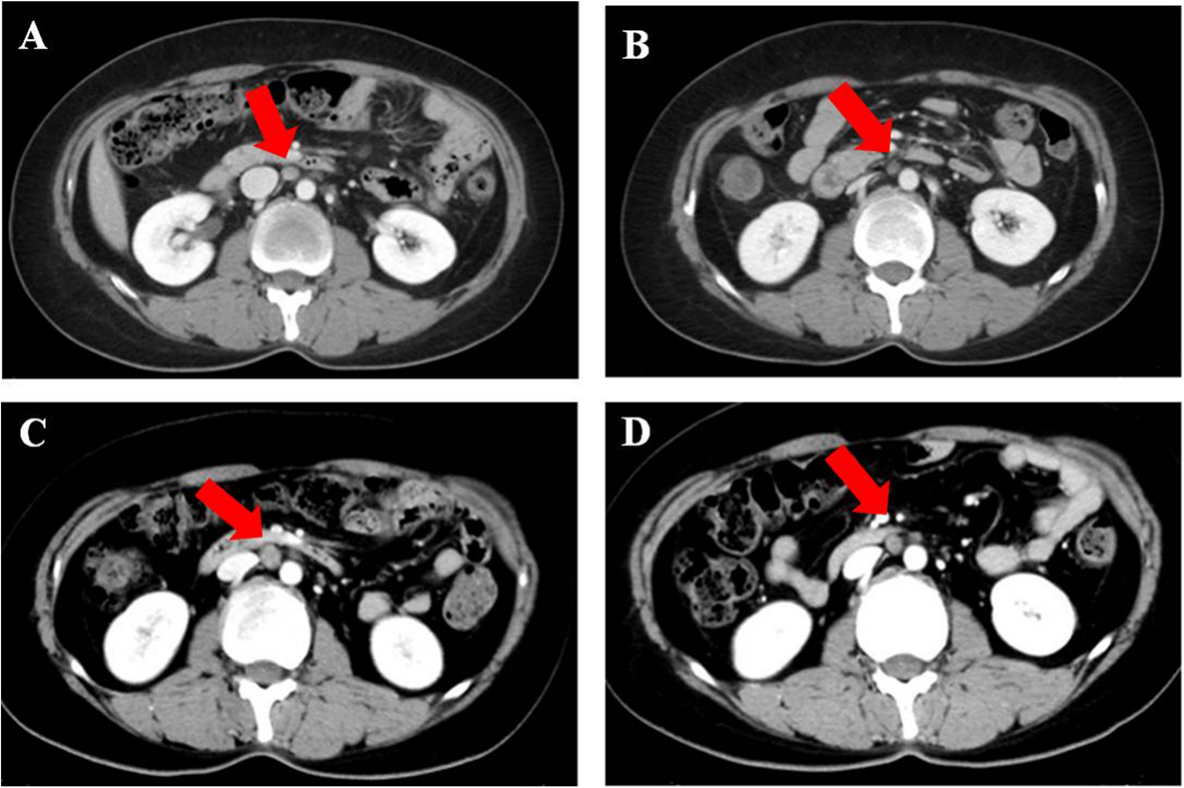
The metastatic para-aortic lymph node pointed with arrow. Notes: **a** recurrence before chemotherapy. **b** partial response after chemotherapy/pretreatment of Olaparib. **c** progressive disease after using Olaparib. **d** partial response after chemotherapy

Patient 4 was a 42-year-old woman with PSR ovarian cancer who underwent a cytoreduction to no macroscopic residual disease (total abdominal hysterectomy, bilateral salpingo-oophorectomy, omentectomy, metastases excised in the uterus-rectum-fossa and surface of the rectum) due to a pelvic mass in July 2013 that was classified as IIIc high-grade serous adenocarcinoma. Then, the patient was treated with paclitaxel plus carboplatin for 8 cycles. In the following 5 years, the patient relapsed five times, and the PFI was more than 6 months every time. In December 2018, the latest recurrence with multiple intrahepatic metastases and enlarged lymph nodes in the retroperitoneum and right pelvis cavity was confirmed by CT (Figs. 6a, and 7a). She was evaluated to have achieved PR according to RECIST1.1 and refused further chemotherapy after 4 cycles of paclitaxel plus carboplatin (Figs. 6b, and 7b). She received olaparib (300 mg orally twice daily) as maintenance treatment because she harbored a germline BRCA1 pathologic mutation. The patient achieved SD after taking olaparib for only 3 months. At the fourth routine follow-up, the abdominal metastasis, intrahepatic metastasis retroperitoneal lymph nodes, and nodules in the lungs and right pleura were larger than before, and very mild ascites was detected in the pelvic cavity along with increasing CA125 levels (Figs. 5, 6c and 7c). Olaparib maintenance therapy was ineffective. After 3 cycles of paclitaxel plus carboplatin chemotherapy, she was assessed as achieving PR again (Figs. 6d and 7d).

**Fig. 8:**
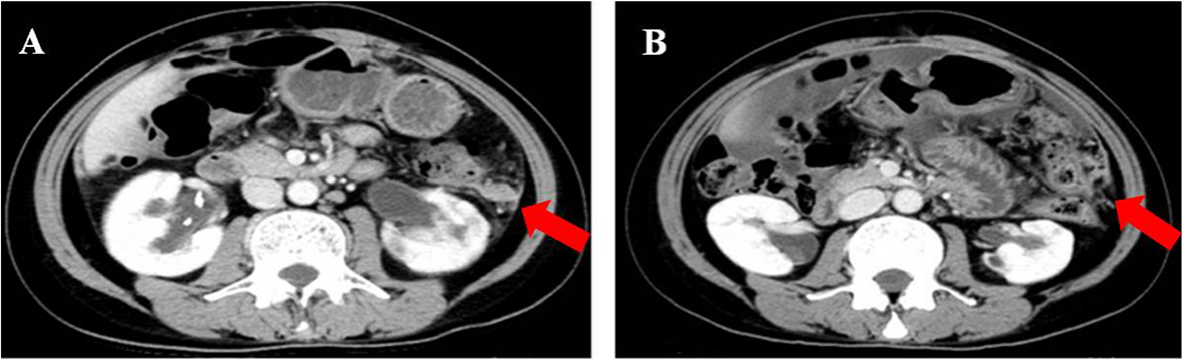
The abdominal metastasis pointed with arrow. Notes: **a** recurrence before chemotherapy. **b** partial response after chemotherapy/pretreatment of Olaparib

**Fig. 9:**
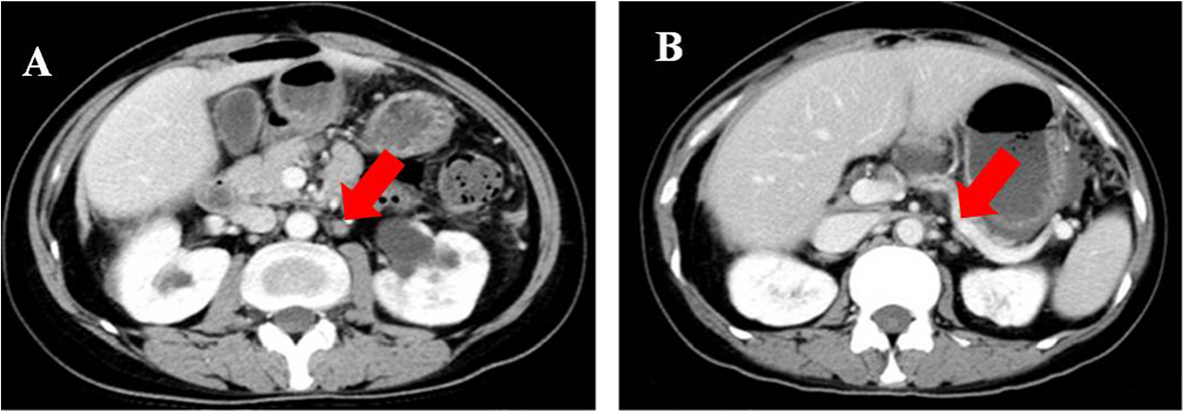
The metastatic para-aortic lymph node pointed with arrow. Notes: **a** recurrence before chemotherapy. **b** partial response after chemotherapy/pretreatment of Olaparib

**Fig. 10:**
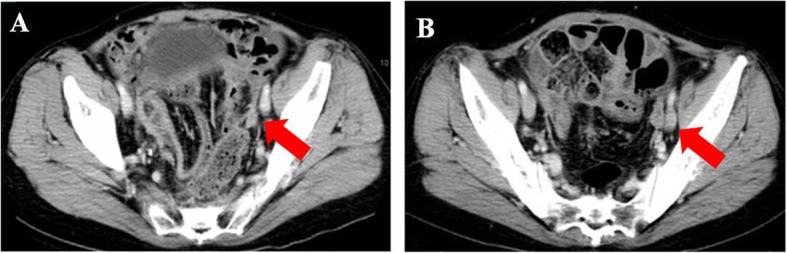
The metastatic in right pelvic pointed with arrow. Notes: **a** recurrence before chemotherapy. **b** partial response after chemotherapy/pretreatment of Olaparib

**Fig. 11:**
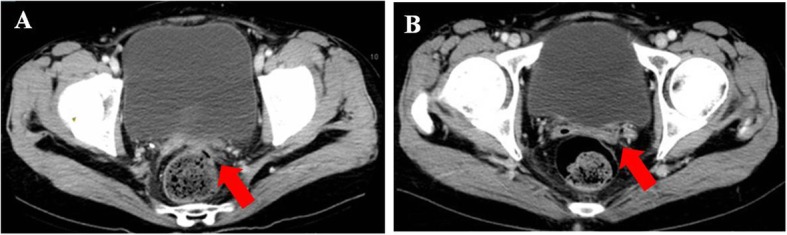
The metastatic vaginal stump pointed with arrow. Notes: **a** recurrence before chemotherapy. **b** partial response after chemotherapy/pretreatment of Olaparib

**Fig. 12:**
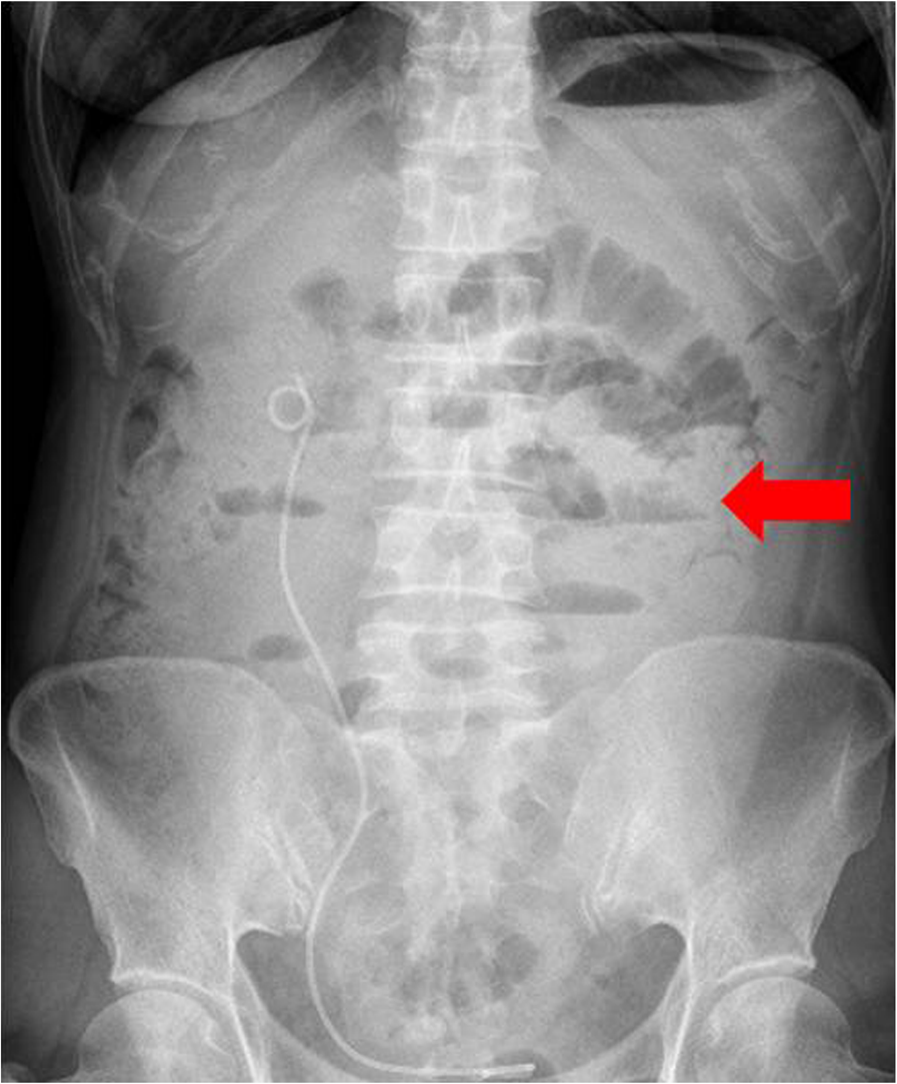
Abdominal plain film of incomplete intestinal obstruction. Notes: progressive disease after using Olaparib

Patient 5 was a 50-year-old woman with recurrent ovarian cancer. In September 2015, she underwent a cytoreduction to no macroscopic residual disease (total abdominal hysterectomy, bilateral salpingo-oophorectomy, omentectomy, appendectomy, metastases excised on the surface of the rectum) due to a pelvic mass and was diagnosed with stage IIIc ovarian endometrioid adenocarcinoma. She received 6 cycles of paclitaxel and carboplatin as first-line therapy. In the 10th month after the initial treatment, the patient relapsed and received the same chemotherapy regimen for 6 cycles. She achieved clinical complete response. However, 3 months later at the first follow-up, multiple metastases in the pelvic and abdominal cavities were observed (Figs. 8a, 9a, 10a and 11a). She underwent another round of salvage chemotherapy and was evaluated to have achieved PR (Figs. 8b, 9b, 10b and 11b). Meanwhile, the patient underwent genetic testing and was confirmed to harbor a germline BRCA2 pathologic mutations. The patient began to take olaparib (150 mg orally twice daily) as maintenance therapy. Two months later, the patient was confirmed to progress with abdominal distension and abdominal pain. The abdominal plain film indicated incomplete intestinal obstruction (Fig. 12), and the patient was recommended for radiological evaluation. However, she refused further examination and treatment. The patient passed away after 1 month of supportive care.

## Discussion and conclusion

Most patients with ovarian cancer will experience repeated recurrence and gradually progress from platinum-sensitive to platinum-resistant. Once the disease has evolved to a platinum-resistant status, the survival duration of patients is significantly shortened [11]. This is one of the main characteristics of advanced epithelial ovarian cancer. Secondary cytoreductive surgery, platinum-based chemotherapy, clinical trials, targeted therapy and immunotherapy are commonly recommended to PSR ovarian cancer patients, while platinum-resistant ovarian cancer (PROC) patients are recommended non-platinum-based chemotherapy, clinical trials, targeted therapy and immunotherapy in some circumstances.

PARP inhibitors have modified the traditional treatment modalities of ovarian cancer, including the initial treatment (surgery plus platinum-based chemotherapy) and the post-relapse treatment. Olaparib has been approved as second-line maintenance therapy for PSR ovarian cancer in many regions, such as the United States, some European countries and China [5]. Furthermore, it can still be used for maintenance treatment of newly diagnosed advanced ovarian cancer with BRCA mutations in China, the United States, Europe and Japan. The use of olaparib as monotherapy in germline BRCA-mutated ovarian cancer after third-line chemotherapy has only been approved by the FDA in the United States [12]. The SOLO3 findings [8] presented at the 2019 ASCO meeting showed a statistically significant and clinically relevant improvement in the ORR and PFS values between the olaparib group and the non-platinum-based chemotherapy group in women with heavily pre-treated PSR gBRCAmt ovarian cancer. Additionally, the CLIO study [9] showed that olaparib monotherapy could improve the ORR in patients with PROC after at least one line of chemotherapy, and the therapeutic effect was more significant in BRCAmt patients than in BRCAwt patients. There has been no study of olaparib as second-line monotherapy for first-time recurrent BRCAwt ovarian cancer thus far.

We explored three patients harboring gBRCAwt ovarian cancer with low tumor burden treated with olaparib as second-line monotherapy. Of the three patients, two were assessed as achieving PR at 9 and 7 months, and one was assessed as having SD at 3 months. The other two patients harboring BRCAmt ovarian cancer with high tumor burden were both assessed as having PD in a short time. All patients suffered mild adverse events (AEs), such as anemia, fatigued and decreased appetite, which were consistent with previous studies from a Chinese cohort [13].

In our series, there was an objective response to PARP inhibitors in three gBRCAwt patients with low tumor loads. In contrast, two BRCAmt patients with high tumor loads failed to obtain a response from olaparib. The preliminary results of the OREO study [14] showed that PARPi-resistant ovarian cancer patients with high tumor load were still responsive to PARPi once the tumor burden was reduced by salvage chemotherapy. The results indicated that tumor burden may influence the effect of PARPi. The subgroup analysis of the SOLO1 [15] study showed that patients with primary cytoreductive surgery, R0 resection and CR outcome were more likely to benefit from first-line maintenance of olaparib than those who received intermittent cytoreductive surgery, did not receive R0 resection and achieved PR after adjuvant chemotherapy. These results illustrated that PARPi for first-line maintenance treatment were more effective for patients with low tumor loads than for patients with high tumor loads. PARPi lead to the formation of double-stranded DNA breaks that cannot be accurately repaired in tumors with homologous recombination deficiency owing to the aberrant activation of low-fidelity repair mediated by nonhomologous end joining, a concept known as synthetic lethality [16]. Compared with traditional targeted and chemotherapy drugs, the antitumor effect of PARPi is lower because of synthetic lethality. PARPi are mainly recommended for maintenance treatment of ovarian cancer, that is, to maintain a low tumor load in ovarian cancer.

Additionally, the present series detected that PARPi as second-line monotherapy were also effective for patients with low tumor loads. Despite the therapeutic effect of olaparib in BRCAwt ovarian cancer with low tumor load, disease progression was found in BRCAmt ovarian cancer with high tumor load in our previous observations and in this study [17]. The better short-term efficacy of the three patients was possibly related to the lower tumor load, which may influence the heterogeneity of tumor cells. Heterogeneity is one of the genetic characteristics of tumors. The tumor cells in patients are in a sustained and rapid evolution state because of the increasing genetic heterogeneity caused by abnormal repair systems, rapid cell proliferation, microenvironmental stress, and so on. The existence of heterogeneity in tumor cell clones has been confirmed to play an important role in drug resistance, metastasis and recurrence. When the tumor burden is low, the heterogeneity of the tumor is at a low level, and the mechanisms of developing drug resistance is mainly branch evolution and linear evolution by DNA damage accumulation. When the tumor burden is high, the level of heterogeneity increases, and drug resistance develops via convergence or horizontal evolution by molecular crosstalk. PARPi-resistant clones have been shown to be affected by the tumor burden [18, 19]. Tumor burden may be another important biomarker for PARPi. There are no standards for assessing low versus high tumor load. The EORTC55971 study verified that a 5 cm diameter was the cut-off for determining the level of tumor burden [20]. The appropriate cut-off point for high versus low tumor load for PARPi needs to be demonstrated by further prospective clinical studies.

PARPi and homologous recombination deficiency (HRD) can produce synthetic lethal effects that lead to cell death in tumor cells [21]. Homologous recombination (HR) is a complex process involving many gene products. In addition to BRCA, mutations of other HR pathway genes (such as MRE11, RAD50, NBS1, ATM, CtlP, PALB2, BRCA2, RAD51, and RPA) can also result in HRD [22]. Several clinical trials have also demonstrated that patients with HRD can benefit from PARPi [23, 24]. Platinum-sensitive tumor cells are likely to have HRD and thus be sensitive to PARPi [25]. The better efficacy of olaparib in BRCAmt ovarian cancer than in BRCAwt ovarian cancer may be related to the PSR status. Another possibility is that all three patients with low tumor loads may have harbored HRD via mechanisms other than alterations in BRCA that made them sensitive to PARPi. However, two BRCAmt ovarian cancer patients with high tumor loads did not benefit from PARPi, suggesting that PARPi may also be ineffective for HRD ovarian cancer with high tumor load. This needs to be confirmed by further observations.

The QUADRA study showed that a 2/3 dose of niraparib had no significant impact on efficacy [24]. In our series, two patients with low tumor load were given a dose of 150 mg bid by themselves. One patient stopped taking the drug for a period of time owing to erysipelas and reduced the dose for 1 month. After taking the drug for 9 months, the patient was assessed as achieving PR. Another patient received olaparib 150 mg bid for 3 months and was assessed as having SD. Whether olaparib has similar efficacy after reduction and whether the dose suitable for Chinese patients can be adjusted according to body mass index (BMI) and the area under curve (AUC) of the Chinese population remain to be confirmed by further research.

One patient with ovarian carcinosarcoma also achieved PR after exploratory therapy, suggesting that olaparib probably has an effect on carcinosarcoma patients and may be related to the tumorigenesis of carcinosarcoma. Previous studies found that epithelial and mesenchymal cells were responsible for the proliferation and differentiation of common pluripotent stem cells in the genesis of carcinosarcoma [26–28].

In conclusion, the present study found that relapsed gBRCAwt ovarian cancer with lower tumor load might also respond to olaparib monotherapy. Furthermore, short-term efficacy and manageable adverse events were observed in these low tumor load cases with gBRCAwt disease, while the high tumor load cases with BRCAmt disease did not benefit from olaparib. Low tumor load may be another potential marker to predict the effect of PARP inhibitors. It will be interesting to view further research on PARP inhibitor monotherapy in relapsed ovarian cancer with low tumor load.

## Data Availability

We would not share the data and material used in this manuscript, because we need them for further research.

## Abbreviations

HTL: High tumor load
LTL: Low tumor load
PARP: Poly adenosine diphosphate ribosome polymerase
PFI: Platinum-free-interval
PSR: Platinum-sensitive relapsed
RECIST: Response Evaluation Criteria in Solid Tumors
